# Lipidomics Profiling of Patients with Low Bone Mineral Density (LBMD)

**DOI:** 10.3390/ijms231912017

**Published:** 2022-10-10

**Authors:** Shereen M. Aleidi, Mysoon M. Al-Ansari, Eman A. Alnehmi, Abeer K. Malkawi, Ahmad Alodaib, Mohamed Alshaker, Hicham Benabdelkamel, Anas M. Abdel Rahman

**Affiliations:** 1 Department of Biopharmaceutics and Clinical Pharmacy, School of Pharmacy, The University of Jordan, Amman 11942, Jordan; 2 Department of Botany and Microbiology, College of Science, King Saud University, Riyadh 11451, Saudi Arabia; 3 Metabolomics Section, Department of Clinical Genomics, Center for Genomics Medicine, King Faisal Specialist Hospital & Research Center (KFSHRC), Zahrawi Street, Al Maather, Riyadh 11211, Saudi Arabia; 4 Department of Chemistry and Biochemistry, The University of Quebec at Montreal, Montreal, QC H3C 3P8, Canada; 5 Department of Family Medicine and Polyclinic, King Faisal Specialist Hospital & Research Center (KFSHRC), Zahrawi Street, Al Maather, Riyadh 11211, Saudi Arabia; 6 Proteomics Resource Unit, Obesity Research Center, College of Medicine, King Saud University, P.O. Box 2925 (98), Riyadh 11461, Saudi Arabia; 7 Department of Biochemistry and Molecular Medicine, College of Medicine, Al Faisal University, Riyadh 11533, Saudi Arabia

**Keywords:** lipidomic, bone mineral density (BMD), osteoporosis, osteopenia, mass spectrometry

## Abstract

The relationship between lipid metabolism and bone mineral density (BMD) is still not fully elucidated. Despite the presence of investigations using osteoporotic animal models, clinical studies in humans are limited. In this work, untargeted lipidomics profiling using liquid chromatography-mass spectrometry (LC-MS) analysis of human serum samples was performed to identify the lipidomics profile associated with low bone mineral density (LBMD), with a subsequent examination of potential biomarkers related to OP risk prediction or progression. A total of 69 participants were recruited for this cohort study, including the osteoporotic group (OP, *n* = 25), osteopenia group (ON, *n* = 22), and control (Ctrl, *n* = 22). The LBMD group included OP and ON patients. The lipidomics effect of confounding factors such as age, gender, lipid profile, body mass index (BMD), chronic diseases, and medications was excluded from the dataset. The results showed a clear group separation and clustering between LBMD and Ctrl (Q^2^ = 0.944, R^2^ = 0.991), indicating a significant difference in the lipids profile. In addition, 322 putatively identified lipid molecules were dysregulated, with 163 up- and 159 down-regulated in LBMD, compared with the Ctrl. The most significantly dysregulated subclasses were phosphatidylcholines (PC) (*n* = 81, 25.16% of all dysregulated lipids 322), followed by triacylglycerol (TG) (*n* = 65, 20.19%), and then phosphatidylethanolamine (PE) (*n* = 40, 12.42%). In addition, groups of glycerophospholipids, including LPC (7.45%), LPE (5.59%), and PI (2.48%) were also dysregulated as of LBMD. These findings provide insights into the lipidomics alteration involved in bone remodeling and LBMD. and may drive the development of therapeutic targets and nutritional strategies for OP management.

## 1. Introduction

Osteoporosis (OP) is a chronic degenerative metabolic bone disease characterized by decreased bone mineral density (BMD), which increases bone fragility and fracture risk [1,2]. This disease is age-related and affects women, particularly postmenopausal, more than men, and is now considered a major public health problem worldwide [3]. It limits patients’ mobility, and its medical and care management is associated with a high social and economic cost [4,5]. 

The pathophysiological basis of OP is an imbalance in the bone remodeling process. Its development is associated with increased bone resorption of osteoclasts, decreased bone formation of osteoblasts, and, consequently, bone loss [3,6]. Lipids are a group of heterogeneous and structurally diverse compounds, including glycerophospholipids, sphingolipids, and sterols. Lipid biological functions include forming cell membranes, storing energy, intracellular signaling, and local hormonal regulation [3]. Emerging research has highlighted the biological roles of lipids and their derivatives as important mediators in bone physiology [3,7]. For example, palmitic saturated fatty acid has been reported to disrupt osteoblast function and survival [8,9]. On the other hand, certain unsaturated fatty acids, including palmitoleic acid, oleic acid, and linoleic acid, have been found to enhance osteoblast differentiation, mineralization capability, and survival [8,10,11] and also to inhibit osteoclast differentiation and function [12,13]. Therefore, it is important to understand the roles of lipids and their derivatives in regulating bone cell balance. Any perturbations in their signaling pathways might result in bone pathologies, such as osteopenia (ON) and the severe form OP [3]. 

Lipidomics, a branch of metabolomics, is a comprehensive lipid-specific tool to analyze lipid molecular classes and provide insight into lipid profiling and understanding disease-associated lipid metabolism [14]. It is based on electrospray ionization high-resolution mass spectrometry (ESI-MS), widely used in lipid-mediated signal transduction and lipid metabolism [14]. Evidence has shown that lipid metabolism is associated with bone metabolism [3,7,15]. Several factors can interfere with bone lipid metabolism, particularly bone marrow cellular balances. These include age, gender, menopause, calcium, vitamin D levels, metabolic disorders, hormonal therapy, and glucocorticoid drugs, [16,17,18]. These factors can affect bone marrow cells and indicate an increase in bone remodeling, leading to low bone density (LBMD). 

Several studies have reported an association between lipid metabolite alterations and LBMD in animal models [14,17] and patients with OP [19,20,21]. However, there is a need for systemic lipidomics studies in humans, both males and females, excluding the confounding factors that might affect lipid metabolism in bone. Therefore, this study aims to identify lipid classes associated with LBMD in patients diagnosed with ON and OP compared with controls. This study would provide insights into the lipidomics changes involved in bone remodeling and LBMD and help to predict new possible biomarkers for OP, therapeutic perspectives, and nutritional strategies for OP management.

## 2. Results

### 2.1. Clinical Characteristics and Demographics of the Study Population

Table 1 presents the clinical characteristics and demographics of the study population. Considering the DXA data, 31.88% of the participants had normal BMD (Ctrl), and 31.88%, and 36.23% were diagnosed with ON and OP, respectively. More than half of the participants were females, and 98% of them were menopausal. The prevalence of ON and OP increased with age. Moreover, the LBMD groups (ON and OP) had significantly lower lumbar and femoral T-scores, fasting blood glucose (FBG), triglyceride (TG), and cholesterol levels, compared with the Ctrl group (Table 1). Additionally, the OP group had a significantly lower lumber T-score than the ON group, as shown in Table 1. 

### 2.2. The Lipidomic Analysis and Exclusion of Confounders-Associated Lipids

**Figure 1:**
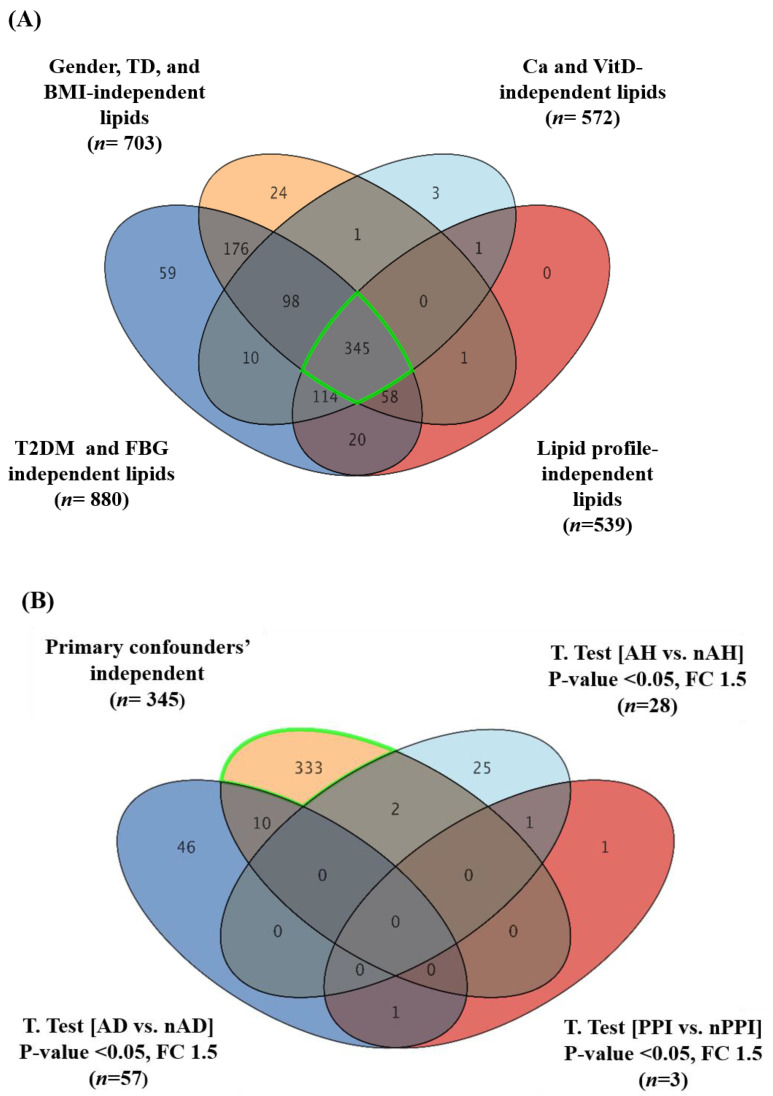

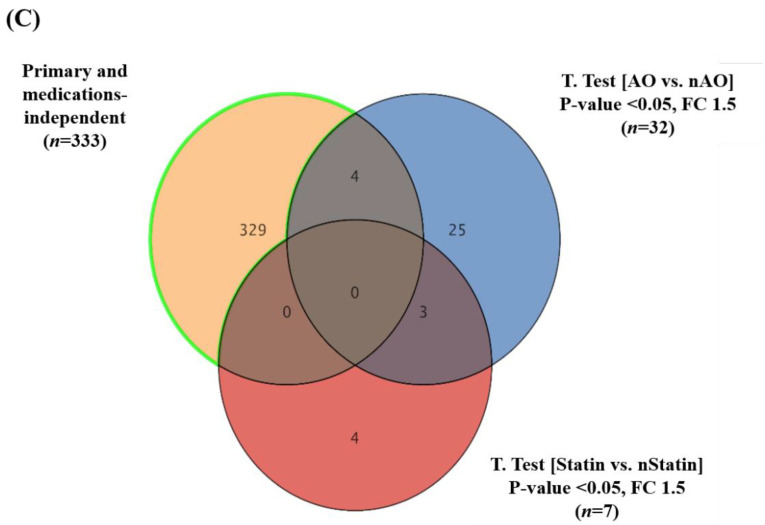
A sequential excluding of the study confounder- and medication-dependent lipids. (**A**) A Venn diagram illustrating the common set of lipids dysregulated due to LBMD (*n* = 345) after excluding the significant confounder effects including gender, thyroid disease (TD), and body mass index (BMI) (*n* = 703) T2DM and FBG (*n* = 880)], patients’ lipid profiles (*n* = 539), and patients’ Ca and Vitamin D3 levels (*n* = 572), using a two-way ANOVA with FDR corrected *p*-value cut-off = 0.05. (**B**) A Venn diagram further excludes the common anti-diabetic (AD) (*n* = 57), proton pump inhibitor (PPI) (*n* = 3), and antihypertensive (AH) (*n* = 28) medication-related lipids, using a moderate *t*-test and considering fold change (FC 1.5) and cut-off *p*-value < 0.05. (**C**) A Venn diagram further excludes other drugs, anti-osteoporotic (AO) and statins, effects from the determined lipids (*n* = 333), using a moderate *t*-test and considering fold change (FC 1.5) and cut-off *p*-value < 0.050. Ultimately, 329 were identified as confounder- and medication-independent lipid molecules.

Following a consideration of the clinical evaluation and demographics of the participants, a group of confounding factors was identified. These included gender, body mass index (BMI), vitamin D3, calcium, fasting blood glucose (FBG), and lipid profiles. These confounders were significantly different between LBMD patients and controls. Furthermore, the presence of metabolic diseases such as type 2 diabetes mellitus (T2DM) and thyroid disease (TD) in the participants were considered confounding factors. In addition, the effect of different medications taken by LBMD patients was considered in this study. Lipid molecules associated with these confounding factors (dependent lipids) were excluded from the analysis. The determination of lipid molecules that are not affected by confounding factors (independent lipids) would enhance the validity of the results in attributing the alteration in the levels of detected lipid molecules to the disease-causing process leading to LBMD in the study population. A Venn diagram analysis using a two-way ANOVA with FDR corrected *p*-value (FDRp) cutoff = 0.05 was carried out to determine confounders’ independent lipids, Figure 1 and Supplementary Figures S1 and S2. A two-way ANOVA with FDR corrected *p*-value (FDRp) cutoff = 0.05 was carried out for each confounder in the group. Groups of 934 and 898 lipids in LBMD patients were not affected by the presence of primary confounders including body mass index (BMI), gender, and Thyroid disease (TD) (Supplementary Figure S1A). A Venn diagram analysis between the two-way ANOVA comparisons resulted in 703 lipids being associated mainly with LBMD after excluding BMI, gender, and thyroid-related lipids (BMI-Gender-TD independent, Supplementary Figure S1A). Similarly, 880 lipids were associated with LBMD after excluding the type 2 diabetes mellitus (T2DM) and fasting blood glucose (FBG) (T2DM-FBG-independent, Supplementary Figure S1B), and 539 lipids were associated with LBMD, regardless of the lipid profile of the patients (Cholesterol-LDL-TG-HDL- independent, Supplementary Figure S1C). In addition, 572 lipids were determined as independent of the levels of calcium and vitamin D3 in LBMD patients (Supplementary Figure S1D). Overlapping of the groups of confounders’ independent lipids revealed 345 lipids were primarily connected to LBMD without the effect of confounders, Figure 1A. 

Moreover, dysregulated lipids resulting from the effect of different medications taken by LBMD patients were determined and excluded from the study’s primary confounders’ lipidomic profile shown in Figure 1 (*n* = 345 lipids). Using a moderate t-test and considering fold change with cutoffs 0.05 (*p*-value < 0.05), and 1.5 (FC), respectively, lipids that were dysregulated by the intake of anti-diabetic medication (AD) (*n* = 57), anti-hypertensive medication (AH) (*n* = 28), proton pump inhibitors (PPI) (*n* = 3), anti-osteoporotic medication (AO) (*n* = 32), and anti-hyperlipidemic (statins) (*n* = 7) were detected, as shown in volcano plots (Supplementary Figure S2). The common medication-related lipids were excluded from confounder-independent metabolites (Figure 1B,C), and therefore 329 lipids were identified as being significantly associated with LBMD, regardless of the effect of confounders and medication, Figure 1C. 

### 2.3. Lipidomics Profiling of LBMD and Control Groups

**Figure 2:**
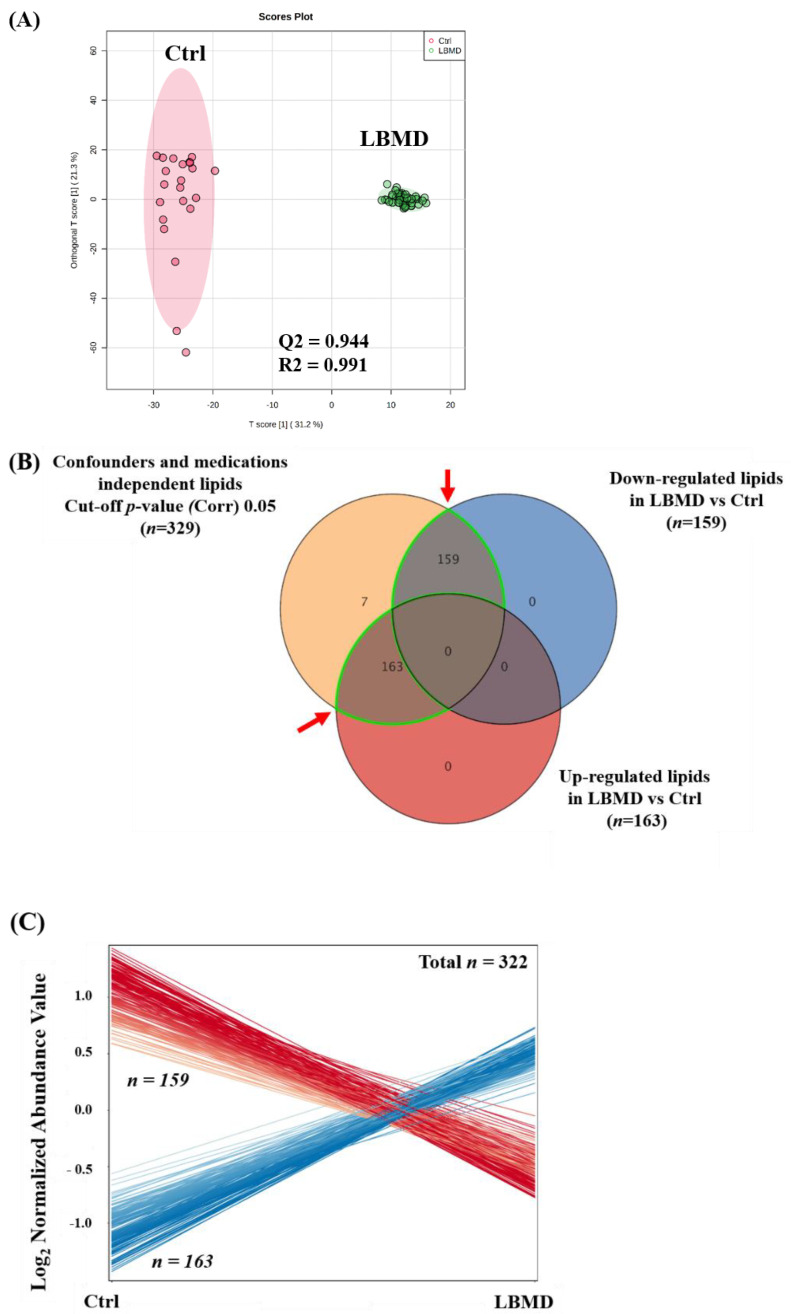

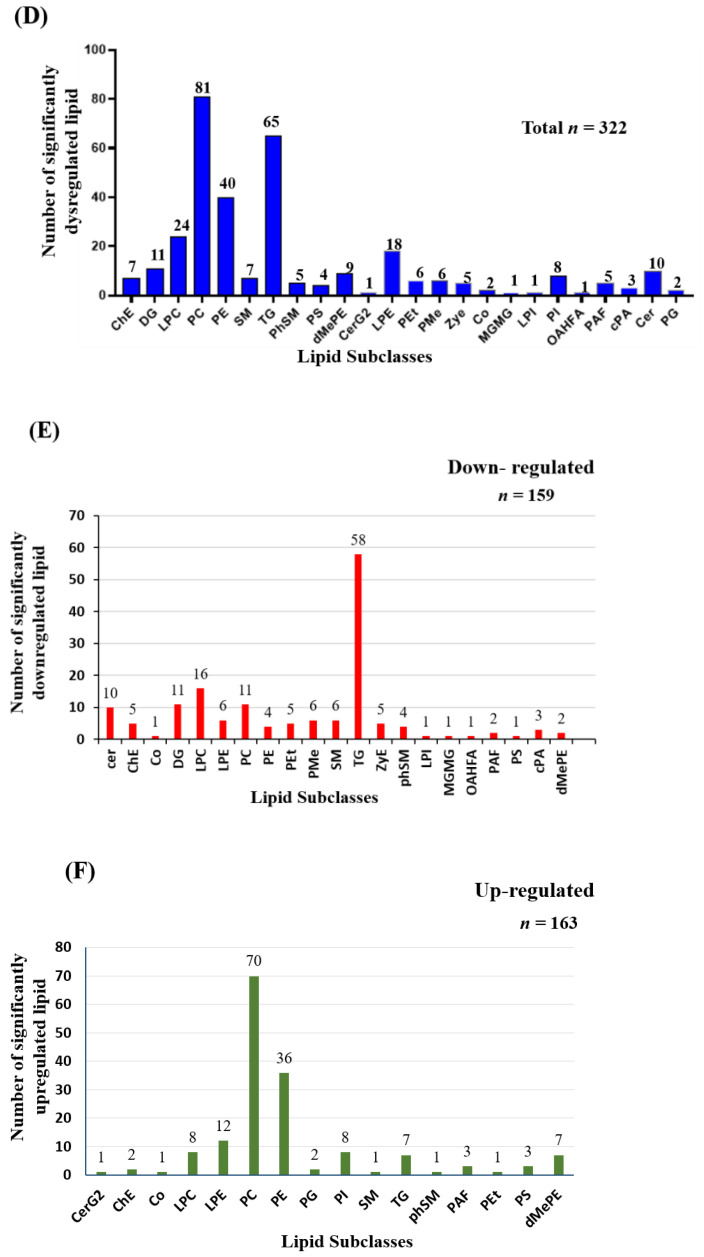
Lipidomics profiling between healthy control (Ctrl) and patients with low bone mineral density (LBMD). (**A**) An Orthogonal Partial Least Squares-Discriminant Analysis (OPLS-DA) module plot illustrates sample clustering and separation between Ctrl versus LBMD groups based on the lipidomics profile, where the robustness of the models was evaluated by the fitness of the model (R^2^Y = 0.944) and predictive ability (Q^2^ = 0.991) values in a larger dataset (*n* = 1000). (**B**) A Venn diagram shows that 322 lipid molecules were dysregulated in LBMD compared with Ctrl; fold change cut-off 2, FDRp-value 0.05. (**C**) The expression of the dysregulated lipid molecules (*n* = 322) was demonstrated, where 163 were upregulated and 159 down-regulated in LBMD compared with Ctrl. (**D**) A bar graph shows the dysregulated lipid molecules (*n* = 322) on subclasses. (**E**,**F**) Bar graphs show the distribution of the down-regulated (*n* = 159) in (**E**) and up-regulated (*n* = 159) in (**F**) lipid molecules in LBMD compared with Ctrl.

Compared with the control group, the lipidomic pattern associated with LBMD was investigated through an orthogonal partial least squares discriminant analysis (OPLS-DA) score plot. Figure 2A shows a clear separation and grouping between the LBMD and control groups (Q^2^ = 0.944, R^2^ = 0.991), reflecting a significant difference in the lipid molecules profile among the compared groups. Considering the panel of confounders and medications-independent lipid molecules (*n* = 329) and based on the fold change cutoff 2 and FDR -corrected *p*-value = 0.05, the levels of 322 lipid molecules were dysregulated as of LBMD, Figure 2B. At the same time, the regulation of 7 lipid molecules was unchanged, Figure 2B. Among these dysregulated lipid molecules (*n* = 322), 163 were up-, and 159 were down-regulated as of LBMD, Figure 2C. The distribution of the significantly dysregulated molecules (*n* = 322) in LBMD compared with control on lipid subclasses is shown in Figure 2D. Overall, twenty-four lipid subclasses were perturbed as of LBMD, and most of these subclasses belonged to glycerophospholipids (phospholipids), glycerolipids, and sphingolipids, Figure 2D. The most significantly altered subclasses were phosphatidylcholines (PC) (*n* = 81, 25.16% of all dysregulated lipids *n* = 322), followed by triacylglycerol (TG) (*n* = 65, 20.19%), and then phosphatidylethanolamine (PE) (*n* = 40, 12.42%). In addition, several glycerophospholipids including lysophosphatidylcholine (LPC) (*n* = 24, 7.45%), lysophosphatidylethanolamine (LPE) (*n* = 18, 5.59%) and phosphatidylinositol (PI) (*n*= 8, 2.48%) were dysregulated as of LBMD, Figure 2D. Interestingly, TGs were the major subclass of downregulated lipids (*n* = 58, 36.48% out of downregulated lipids 159), Figure 2E. PC (*n* = 70, 42.94% out of 163) and PE (*n* = 36, 22.09%) were the most up-regulated subclasses, Figure 2F. The identity of lipid molecules in the subclasses is presented in Supplementary Table S2. The abbreviations of lipid classes are presented in Supplementary Table S3.

### 2.4. Biomarker Evaluation for the Identified Common and Significant Lipids between LBMD and Control

**Figure 3:**
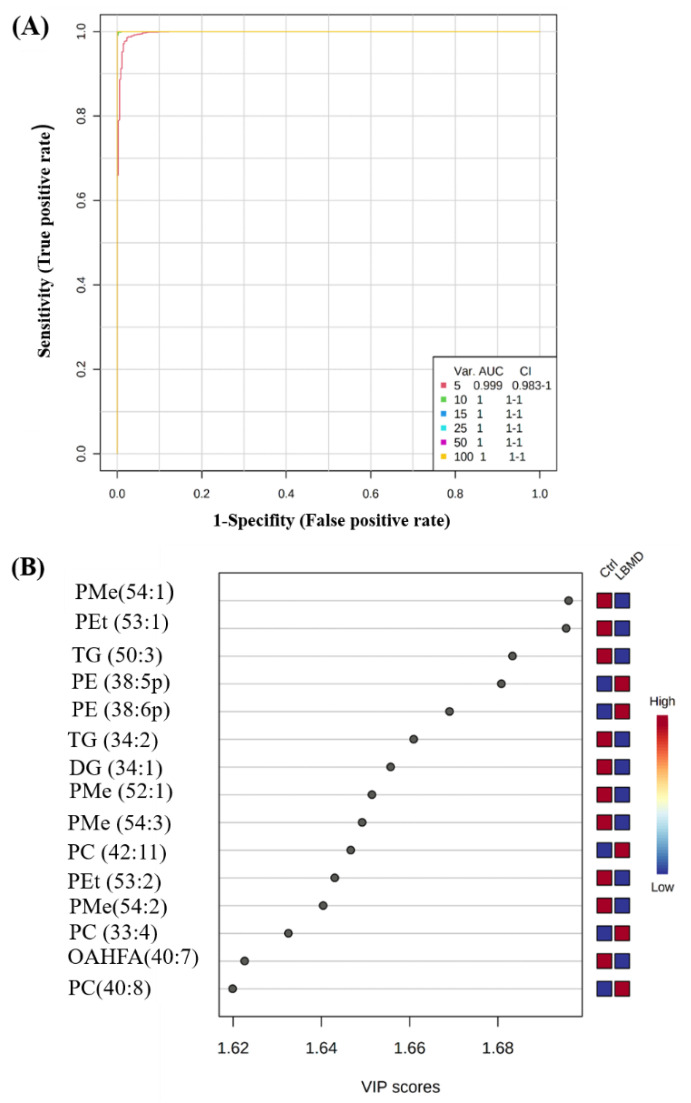

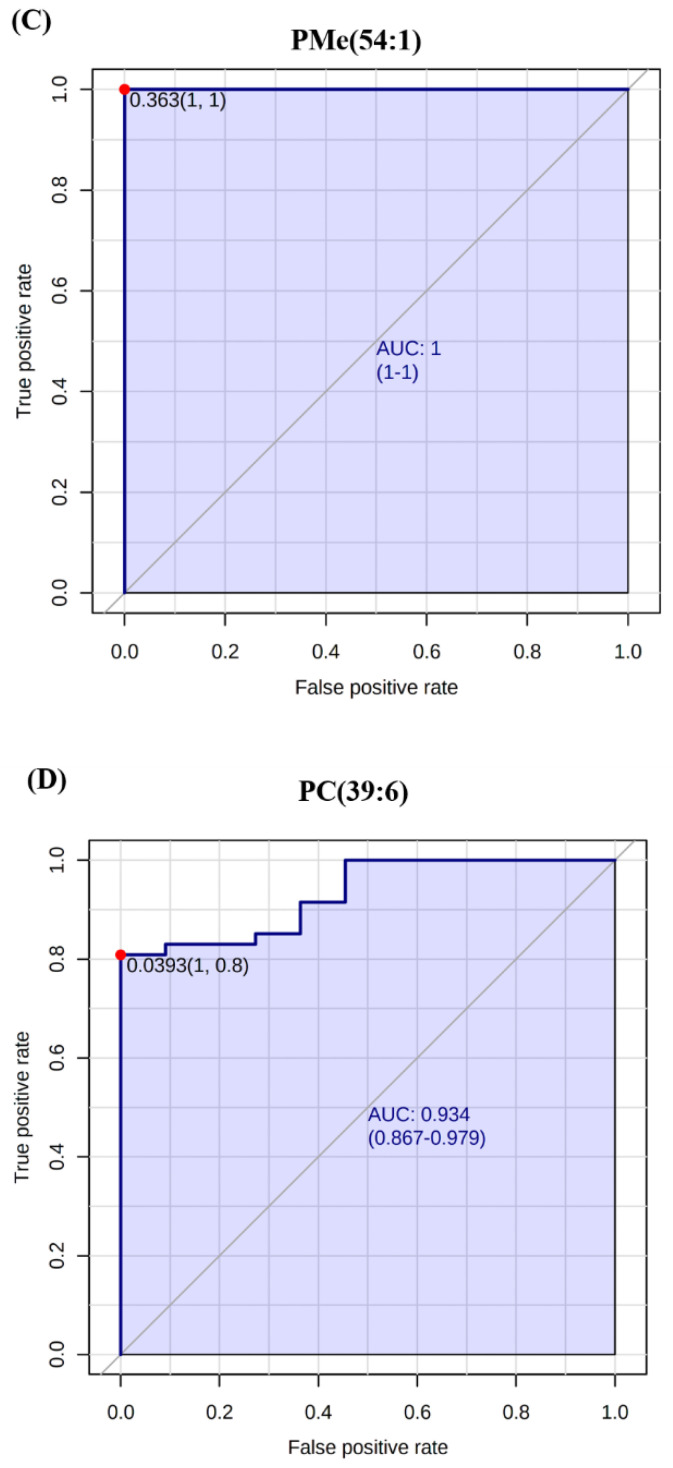

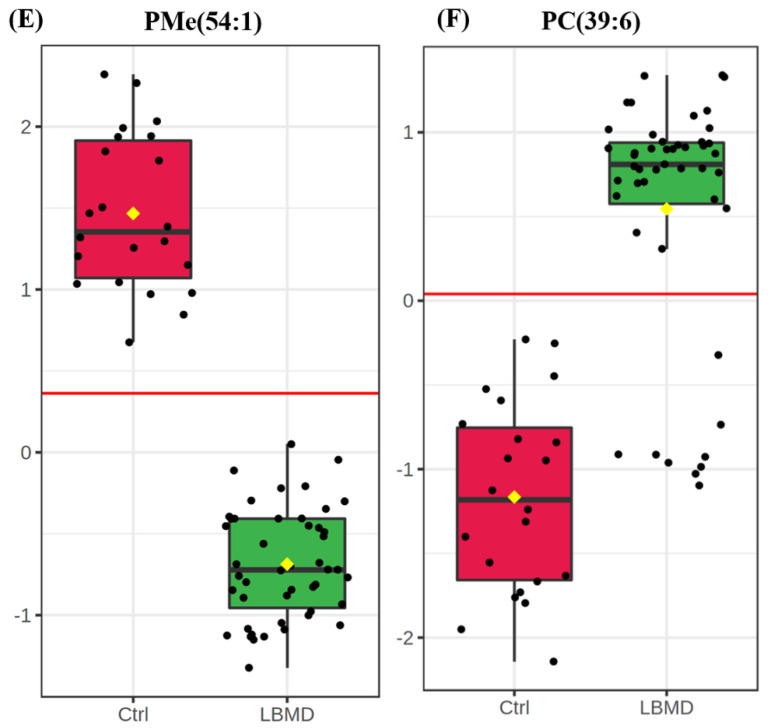
Lipidomic profiling and biomarker evaluation between healthy Ctrl and LBMD patients. (**A**) An exploratory ROC curve generated by the OPLS-DA model, with the area under the curve (AUC) values calculated from the combination of 5, 10, 15, 25, 50, and 100 lipid molecules. (**B**) VIP plot showing the top 15 positively identified lipid molecules. (**C**,**D**) Representative ROC curves for two significantly dysregulated lipid molecules (PMe (54:1), AUC = 1, and Phosphatidylcholines (PC (39:6), AUC = 0.934)). (**E**,**F**) Boxplots for representative significantly dysregulated lipids in LBMD compared with Ctrl, PMe (54:1) is significantly down-regulated in LBMD, *p*-value = 4.54E^−^^29^, where PC (39:6) is significantly up-regulated in LBMD, *p*-value = 1.59E^−^^13^. (PMe: phosphatidylmethanol, PC: phosphatidylcholine.)

A multivariate exploratory receiver operating characteristic (ROC) analysis based on the identified common and significant dysregulated lipids between the LBMD and control (*n* = 322) was generated, using the OPLS-DA model as a classification and feature-ranking method. Combining the top 10 lipid molecules in the exploratory ROC curves indicates the maximum confidence of differentiation and detection of lipids in LBMD versus control, with the area under the curve (AUC) =1 (Figure 3A). The significant features of the positively identified lipid molecules are shown in Figure 3B. The performance of two putatively identified lipid molecules was individually evaluated as showcasing the potential use of lipidomics for biomarker candidate discovery for LBMD. The AUCs for the two putatively identified dysregulated lipid molecules, namely, phosphatidylmethanol (PMe (54:1), AUC = 1)) and phosphatidylcholine (PC (39:6), AUC = 0.934)) are shown in Figure 3C,D. Moreover, as shown in the boxplots (Figure 3E,F), PMe (54:1) was significantly down-regulated, while PC (39:6) was significantly up-regulated in LBMD, compared with the control, (*p*-value = 4.54 × 10^−^^29^, *p*-value = 1.59 × 10^−^^13^, respectively). 

### 2.5. Lipidomics Profiling between ON and OP Groups

**Figure 4:**
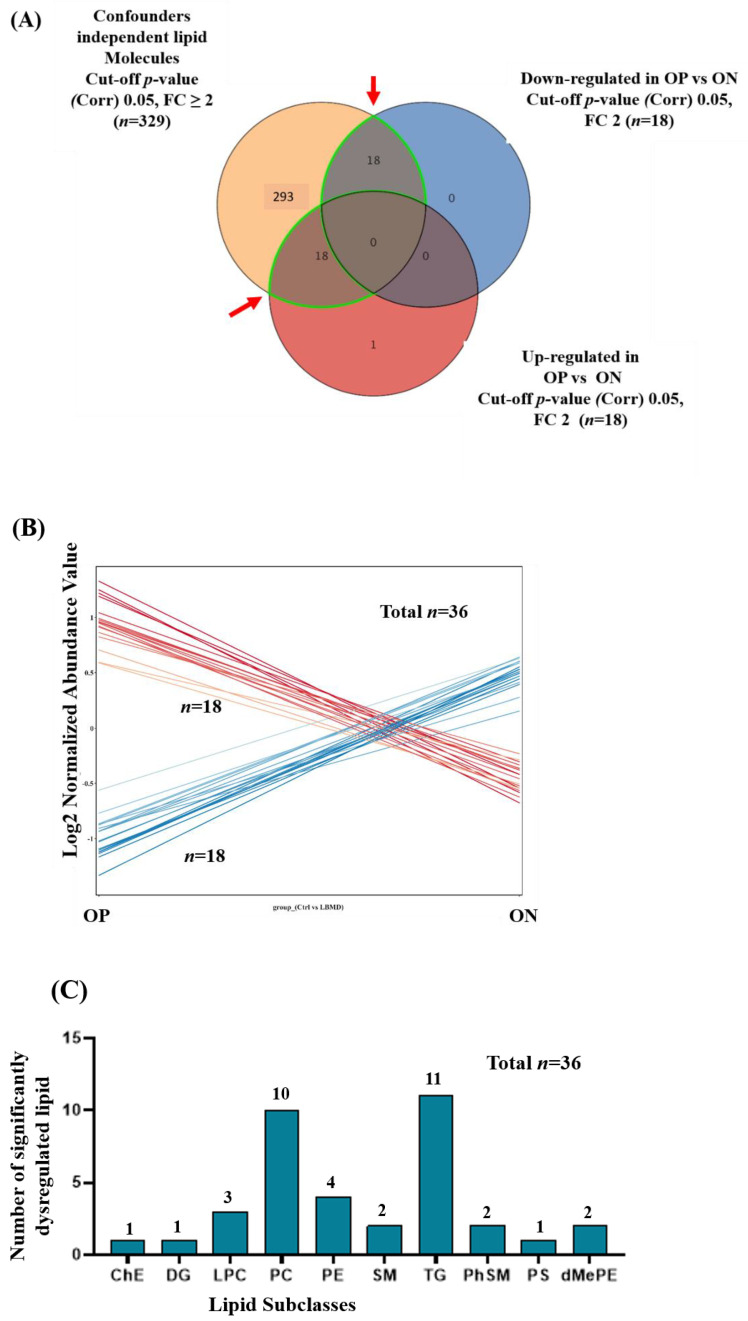

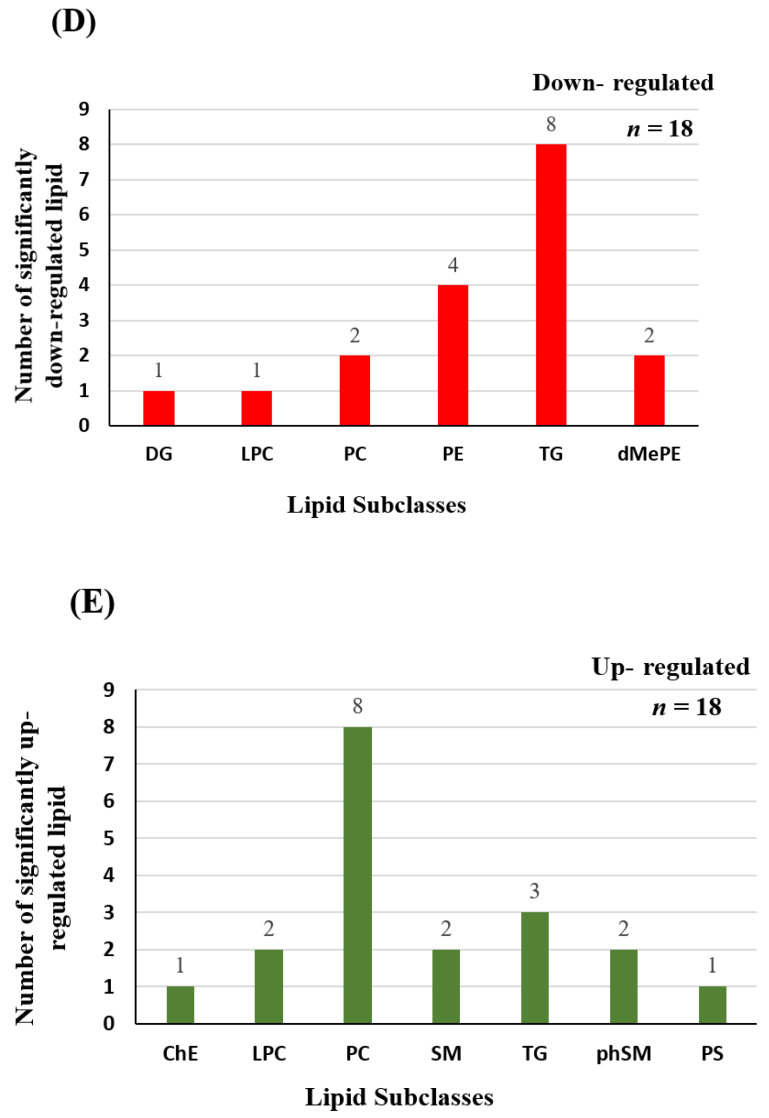
Lipidomic dysregulation associated with OP and ON. (**A**) Venn diagram shows the determination of the dysregulated lipids associated with OP compared with ON from the panel of confounder- and medications-independent lipids (*n* = 329); based on the fold change trend (cutoff ≥2, and <0.8), 36 lipid molecules were dysregulated in OP compared with ON (**B**) The expression of the dysregulated lipid molecules (*n* = 36) was demonstrated, where 18 were up-regulated and 18 were down-regulated in OP, compared with ON. (**C**) A bar graph shows the subclasses’ dysregulated lipid molecules (*n* = 36). (**D**,**E**) Bar-graphs showing the distribution of down- (*n* = 18) and up-regulated (*n* = 18) lipid molecules in subclasses.

Depending on their T-scores, patients with LBMD were classified into ON and OP groups. Considering the clinical characteristics and demographic data presented in Table 1, there were no significant differences between ON and OP patients in any of the mentioned parameters, except for lumber and femoral T-scores and a slight, not significant, difference in height. To determine the potential dysregulated lipid molecules between ON and OP, a Venn diagram analysis was performed, considering the significant and confounder independent lipids (*n* = 329) and based on an FDR-corrected *p*-value < 0.05 and FC ≥2 or <0.8. Figure 4A,B show that 36 lipid molecules were significantly dysregulated between OP and ON groups, divided into 18 up- and 18 down-regulated in OP, compared with the ON. The distribution of the significantly dysregulated molecules (*n* = 36) in OP compared with ON on lipid subclasses indicated that ten subclasses were dysregulated, as shown in Figure 4C. The pattern of lipid molecules distribution in the subclasses was similar to that between the LBMD and control, as the number of significantly dysregulated lipids was the highest for TG, PC, and PE (*n* = 11, 30.33% out of 36 dysregulated lipids, *n* = 10, 27.78%, and *n* = 4, 11.11%, respectively). Furthermore, the distribution of down- and up-regulated lipid molecules in the subclasses is shown in Figure 4D,E, respectively. The most down-regulated subclasses were TG (*n* = 8, 44.44% out of 18 down-regulated lipids), followed by PE (*n* = 4, 22.22%). In contrast, PC were the most upregulated subclasses (*n* = 8, 44.44% out of 18 up-regulated lipids) in OP, compared with ON. The identity of lipid molecules in the subclasses is presented in Supplementary Table S4.

## 3. Discussion 

In this study, an untargeted lipidomics profiling using LC-MS analysis of human serum samples was conducted, to explore the lipidomics profile associated with LBMD and the subsequent identification of potential biomarkers related to OP risk prediction or progression. Some studies in the literature have examined the molecular mechanisms correlated with cholesterol-mediated bone deterioration (reviewed in [15]). High total cholesterol levels are negatively associated with BMD, while treatment with cholesterol-lowering drugs (statins) promotes bone production and thus enhances BMD [22,23]. Clinical studies investigating the changes in lipid metabolism and the levels of lipid molecules associated with LBMD in humans are limited. To the best of our knowledge, this is the first lipidomics study including patients diagnosed with ON and the more severe progressive OP, and investigating the lipidomics profiles of LBMD patients versus healthy controls. Moreover, the impact of confounding factors on BMD in this study, including gender, BMI, patients’ lipid profiles, and chronic diseases, were considered and excluded from the analysis dataset.

### 3.1. Lipidomics Profiling of LBMD and Control Groups

The results revealed that 329 putatively identified lipid molecules were independent of the determined confounders, and utilized for studying the lipidomics profile associated with LBMD. Among these lipid molecules, 322 were dysregulated, with 163 up- and 159 down-regulated in LBMD, compared with controls. Classifying these lipid molecules into subclasses indicated that PC, TG, and PE were the most significantly dysregulated as of LBMD. In addition, a group of glycerophospholipids, including LPC, LPE, and PI, were also dysregulated as of LBMD. These findings were in line with two recent lipidome-wide association studies of shared early lipidomic biomarkers of subclinical OP and atherosclerosis comorbidity [21,24]. Based on their results, the most significant lipid classes associated with OP were glycerolipids (such as TG), glycerophospholipids (such as PC), and sphingolipids [21,25]. Moreover, studies that used an LC-MS untargeted metabolomics approach also reported a change in the levels of different lipid classes, including glycerophospholipids, glycerolipids, and sphingolipids in postmenopausal women with LBMD [20,26,27]. In addition, consistent with our findings in human samples, LC-MS untargeted metabolomics analyses of samples of a glucocorticoid-induced OP in an animal model revealed significant changes in glycerolipids, glycerophospholipids such as lysophospholipids, and sphingolipids such as ceramides [14,17]. These accumulating findings of our and other research groups imply that lipids play a key role in bone metabolism and mineralization.

Furthermore, our results showed that PC and PE were among the most abundant upregulated lipid subclasses in the LBMD group. Both PC and PE belong to the glycerophospholipids class and are important components of the lipid bilayer in cell membranes. They have a choline and ethanolamine head, respectively, in their structures. PCs are essential in cell signaling, energy storage, and glycerophospholipids metabolism [28]. PEs are involved in cell–cell membrane fusion, mitochondrial stability, and autophagy [28]. In vitro studies have shown that the cellular content of glycerophospholipids, particularly PEs, is associated with osteoclastogenesis, and increases during the osteoclast differentiation [29]. In OP, there is an increase in osteoclast differentiation and activity associated with a decrease in osteoblast differentiation and mineralization [30]. Therefore, this might justify the increased levels of PE in the LBMD group in this study.

In this study, LPCs, a metabolite of PCs, were among the dysregulated lipids of LBMD. Omics studies which used osteoporotic animal models reported altered levels of LPCs [17,31]. It has been shown that LPCs induce the production of reactive oxygen species and promote oxidative stress damage in tissues [32,33]. This indicates that the increase in oxidative stress might be associated with an increase in bone loss and thus an exacerbation of OP [34]. Overall, our findings of increased glycerophospholipids (PC, PE, and LPC) are due to the process of OP progression.

Interestingly, our results indicated that TGs were among the most abundant downregulated lipids in the LBMD group. TGs are neutral lipids consisting of glycerol attached to three FAs via ester bonds, and act mainly as storage lipids (Fat). Lipids are present in two compartments of the bone; the bone marrow (BM; soft tissue) and the mineralized tissue (MT; the hard part) [3]. Animal studies have investigated the changes in the content of local bone lipids related to OP, using lipidomics profiling of bone tissue in femurs of ovariectomized (OVX) osteoporotic mouse models [14,35]. Both studies reported an accumulation and significant increase of fat content, mainly glycerolipids (primarily TGs), sterol, and Cer in the bone tissue, supporting the hypothesis that OP is characterized by bone loss associated with an increased BM adiposity. In this study, we conducted a lipidomics analysis of the circulating lipids in human serum samples rather than bone tissue. In addition, the analyzed lipid molecules were independent of the effect of patients’ lipid profiles (as detailed in the results Section 4.2). Based on animal studies findings, the decreased levels of TGs in the serum of LBMD patients might be justified by the accumulation of fat (mainly TGs) in the bone tissue during OP. However, this raises the question of whether this process is mediated by the infiltration of blood TGs or the synthesis of TGs by osteocytes. Therefore, further lipidomics analysis of human bone tissue is required to confirm this hypothesis.

### 3.2. Biomarker Evaluation for the Identified Common and Significant Lipids between LBMD and Control

Interestingly, this study’s lipidomics profiling of LBMD patients was consistent with our recent metabolomics analysis [36]. We have shown that the biosynthesis of unsaturated fatty acids (FAs) was significantly dysregulated in the case of LBMD, and the levels of several PCs were upregulated in OP compared with ON patients [36]. In this study, an untargeted comprehensive lipidomics analysis indicated that (phosphatidylcholine PC (39:6)), phosphatidylcholine has two FAs, with a total of 39 carbons and 6 double bonds in the FAs chains, and is significantly increased in LBMD compared with the control. Different in vitro and in vivo studies reported the role of FAs in compromising bone health by affecting osteoblast function and stimulating osteoclastogenesis [37,38]. For example, the saturated FA, palmitic acid (PA, 16:0), has been shown to promote receptor activator of NF-κB ligand (RANKL)-stimulated osteoclastogenesis and also to induce osteoclast differentiation, even in the absence of RANKL [37]. On the other hand, the role of polyunsaturated fatty acids (PUFAs) with a chain length longer than 18 to 20 carbons in bone metabolism depends on the location of the first double bond, which is either at the third (*n* − 3) or the sixth (*n* − 6) carbon from the methyl end, [39]. It has been shown that *n* − 3 PUFAs stimulate osteoblastogenesis by decreasing peroxisome proliferator-activated receptor gamma (PPARγ) expression, thus enhancing osteoblastic activity. At the same time, the *n* − 6 PUFAs suppress the osteoblasts differentiation via increasing PPARγ expression and promote adipogenesis (reviewed in [39]). Taken together, our previous and current findings imply the importance of FAs and lipid subclasses containing FAs (i.e., PCs) in maintaining bone hemostasis and the possibility of considering their levels as potential biomarkers for OP progression.

In this study, confounder-related lipid molecules were excluded from the LBMD-associated profile, using an analytical approach that would enhance the validity of the results by excluding the effect of confounders on the lipidomics profile. However, the putatively identified lipid molecules associated with LBMD still need validation using an independent cohort from different backgrounds. Furthermore, the number of recruited participants in this study was relatively small. Recruiting control participants was challenging, as it required searching for healthy, medically free, and age-matched participants with a normal DXA scan.

## 4. Materials and Methods

### 4.1. Study Population

A total of 69 participants were involved in this cohort study. Participants were recruited from the OP clinic at King Faisal Specialist Hospital and Research Center (KFSHRC), Riyadh, Saudi Arabia, from December 2017 to January 2019. A central dual-energy X-ray absorptiometry (DXA) scan was used for OP diagnosis. Lumbar and femoral T-scores were measured for patients. In line with the world health organization (WHO) diagnostic criteria and the BMD T-score, participants were categorized into three groups. The osteoporotic group (OP, *n* = 25) included patients with BMD T-scores less than −2.5, while patients with T-scores from −1 to −2.5 were included in the osteopenia group (ON, *n* = 22). Participants with a T-score of more than −1.0 were the healthy control group (Ctrl, *n* = 22). Both OP and ON groups were initially considered together as the LBMD group. Participants who were 50 years old and confirmed as diagnosed with either OP or ON were eligible for this study. Participants under 50 and those diagnosed with concomitant chronic infectious arthritis, chronic lung disease, hepatic diseases, cardiovascular diseases, and renal failure, were excluded from this study. In addition, patients on medications such as glucocorticoids or hormonal replacement (estrogen and androgen therapy) were excluded. Participants’ demographic and clinical data were collected from the primary physician, using an approved questionnaire. 

### 4.2. Ethics Statement

All procedures conducted in this study, including human participants, followed the Declaration of Helsinki’s ethical standards and the universal International Conference on Harmonization-Good Clinical Practice (ICH-GCP) guidelines. This study was reviewed and approved by the Institutional Review Board (IRB) at King Faisal Specialist Hospital and Research Center (KFSHRC) (RAC #2180 003), Riyadh, Saudi Arabia. Written informed consent was obtained from all participants.

### 4.3. Lipidomics Analysis

#### 4.3.1. Sample Preparation

Participants’ serum samples were thawed on ice, and 100 uL were transferred with 5 uL of Standard Lysophosphatidylcholines (LPC) (12:0) (125 ug/mL) ino a fresh Eppendorf tube. The mixture was vortexed for 1 min. The lipid molecules were extracted with 1.5 mL of Chloroform/Methanol (*v*/*v* 2:1) and followed 0.5 mL water. The mixtures were vortexed for 1 min, and the slurry spun down at 3000 r.p.m for 10 min at 4 °C. After protein precipitation, the bottom organic layers were transferred to a fresh tube, and the solvent was dried under a nitrogen gas stream. The dry residuals were resuspended with 200 uL isopropanol/methanol (*v*/*v* 1:1). The mixture was vortexed for 1 min and centrifuged at 1200 r.p.m for 10 min at 4 °C. The supernatant was transferred to autosampler vials for LC-MS analysis. Sample preparation and analyses were randomized. A pool containing aliquots of all samples was employed as quality control (QC), where identical QC aliquots were extracted with the samples to control the reproducibility of the extractions and injections. Samples were extracted in polypropylene microcentrifuge tubes and stored in polypropylene autosampler inserts in amber injection vials capped with polytetrafluoroethylene (PTFE)-lined caps. The blanks were extracted using water instead of a sample and methanol instead of an internal standard mixture.

#### 4.3.2. Liquid Chromatography-Mass Spectrometry (LC-MS) Analysis

Two uLs of each sample were injected into a Phenomenex Kinetex C18 column (100 mm × 2.1 mm, 1.7 μm) at Thermo Ultimate 3000 LC system. The lipid molecules were chromatographed in a gradient system (Table S1), where solvent A was acetonitrile/water (*v*/*v* 6:4) containing 10 mmol/L ammonium format, and solvent B was acetonitrile/isopropanol (*v*/*v* 1:9) containing 10 mmol/L ammonium format. The flow rate of the mobile phase was 0.3 mL/min, and the column was maintained at 50 °C. The column eluents were introduced into a Thermo Q Exactive mass spectrometer (City, Country). The mass spectrometer was operated in positive and negative ionization modes with full scan MS at 70,000 resolution and data-dependent MS/MS collected from 200–1200 *m*/*z* at 17,500 resolution. The electrospray ionization source was maintained at a spray voltage of 3 kV at positive ion mode and −2.8 kV at negative ion mode with sheath gas at 35 and auxiliary gas at 15 (arbitrary units). The inlet of the mass spectrometer was held at 350 °C, and the S-lens were set to 50%. The acquisition sequence was randomized for samples and blank extracts, with one QC injection after every 10 injections. Hence, the QC results include extraction and injection replicates of the pooled mixture. The samples were analyzed twice in positive and negative ionization in the same randomized order. A 1.0 min mass re-calibration segment was inserted between the end of each chromatogram and column re-equilibration, during which 0.10 mM sodium formate calibrant solution was infused with a peristaltic pump.

### 4.4. Statistical Analysis

The MS raw data were normalized to the total sample median, and log-transformed and Pareto scaled to provide all the Gaussian distributed signals using MetaboAnalyst V5 (McGill University of Montreal, Montreal, QC, Canada) [39]. A univariate analysis using a volcano plot analysis was performed for each binary comparison, to identify significantly differentially expressed lipid molecules based on a fold-change criterion greater than 1.5 or less than 0.67, with a false discovery rate (FDR) adjusted *p*-value less than 0.05. The x-axis on the volcano plot represents the fold change (FC) between the two comparison groups, while the y-axis represents the *p*-value. Multivariate analysis (orthogonal partial least squares-discriminant analysis (OPLS-DA)) was conducted to identify any clustering or separation between the compared data sets. 

For statistical analysis among the groups, analysis of variance (ANOVA) using post hoc Tukey’s analysis method, with multiplicity-adjusted *p*-values, for each comparison was used. This analysis seemed the best for reducing the probability of making a type 1 error, since it supports the testing of pairwise differences due to the unequal group sizes among the experimental and the control groups as seen in our cohorts. A Pearson similarity test, Hierarchical clustering combined with heat maps, and Venn diagram analyses, including the two-way ANOVA were performed between the study groups, using Multiple Professional Profiler (MPP) software (Agilent Inc., Santa Clara, CA, USA). 

## 5. Conclusions

In this study, we applied comprehensive, untargeted LC-MS lipidomics to identify the altered lipid molecules associated with LBMD in patients diagnosed with either ON or OP, compared with controls with normal BMD. The results of this lipidomics study support the findings of previous studies and highlight the role of different lipids subclasses in OP progression. In addition to TG, glycerophospholipids, including PC, PE, and LPC, were changed in patients with LBMD, suggesting that the abnormal metabolism of glycerolipids and glycerophospholipids are related to osteoporosis. These findings provide insights into the lipidomics alteration involved in bone remodeling and LBMD, and may drive the development of therapeutic targets and nutritional strategies for OP management.

## Figures and Tables

**Table 1:** Clinical characteristics and demographics of the study population (*n* = 69).

	Ctrl		ON		OP	
Total *n* (%)	22 (31.88)		22 (31.88)		25 (36.23)	
parameters	Mean	SEM	Mean	SEM	Mean	SEM
Age (y)	54.82	1.03	64.64 ^§^	1.72	66.16 ^§^	1.78
Gender (F/M)	(13/9)	-	(15/7)	-	(24/1)	-
Menopause * (Yes/No)	(13/0)	-	(14/1)	-	(24/0)	-
Weight (kg)	85.13	3.63	74.21	3.88	69.23 ^§^	2.86
Height (cm)	162.22	0.02	157.11	0.021	150.68 ^§^ǂ	0.01
BMI (kg/m^2^)	32.21	1.1	30.38	1.84	30.70	1.4
Lumbar t score	0.29	0.24	−1.25 ^§^ ǂ	0.21	−2.62 ^§^	0.12
Femoral t score	0.34	0.29	−1.51 ^§^ ǂ	0.14	−1.93 ^§^	0.13
FBG (mmol/L)	10.2	1.16	6.08 ^§^	0.39	5.87 ^§^	0.41
HDL (mmol/L)	1.00	0.80	1.47 ^§^	0.12	1.42 ^§^	0.09
TG (mmol/L)	1.85	0.15	1.23 ^§^	0.11	1.127 ^§^	0.08
Cholesterol (mmol/L)	5.51	0.23	4.47 ^§^	0.19	4.27 ^§^	0.29
Calcium (mmol/L)	2.24	0.026	2.37 ^§^	0.025	2.33 ^§^	0.02
Albumin (g/L)	37.65	1.14	41.98 ^§^	2.0	42.75 ^§^	0.86
Vitamin D 25 hydroxy (nmol/L)	68.32	7.39	77.64	3.3	86.57	6.05

Abbreviations: ON: osteopenic, OP: osteoporotic, BMI: body mass index, FBG; fasting blood glucose, LDL; low-density lipoprotein, HDL; high-density lipoprotein, TG; triglycerides. Data are presented as mean ± standard error of the mean (SEM); * menopause status in females; ^§^ *p*-value < 0.05 vs. control group; ǂ *p*-value < 0.05 vs. OP group.

## Supplementary Materials

The following supporting information can be downloaded at: https://www.mdpi.com/article/10.3390/ijms231912017/s1.
