# Supplementary material for: Lipidomics Profiling of Patients with Low Bone Mineral Density (LBMD)
Source: Int J Mol Sci. 2022 Oct 10;23(19):12017. doi: 10.3390/ijms231912017 (PMC9570421; doi:10.3390/ijms231912017)
Supplement: Supplementary file 1 [file ijms-23-12017-s001.zip › ijms-1937787-supplementary.pdf]

Supplementary Figure S1 and S2

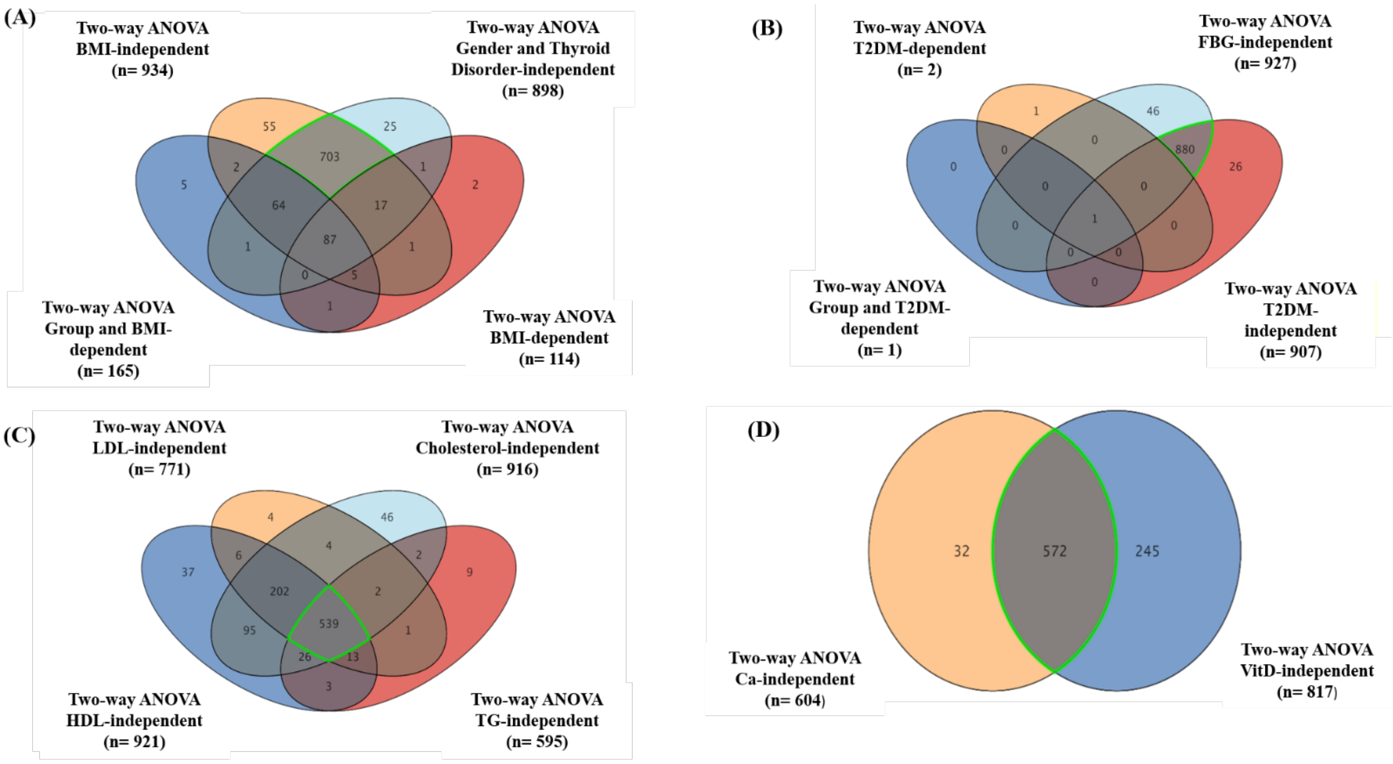

Figure S1: Determination of lipids independent on a group of confounders.

The panels from (A-D) show Venn diagrams demonstrating overlapping and determination of lipids not affected by a group of primary confounders in patients with LBMD, using Two-way ANOVA with FDR corrected p-value cut-off = 0.05. (A) Extraction of lipids (n=703) dysregulated in LBMD patients independently on Gender, BMI, and thyroid disease (TD). (B) Extraction of lipids (n= 880) dysregulated in LBMD patients regardless of the effect of T2DM and FBG. (C) Extraction of lipids (n=539) dysregulated in LBMD patients independently on the lipid profiles. (D) An overlap between Two-way ANOVA comparisons of groups of lipids that are Ca- independent (n= 604) and vitamin D3 independent (n=817) resulted in 572 lipids independent on both LBMD patients.

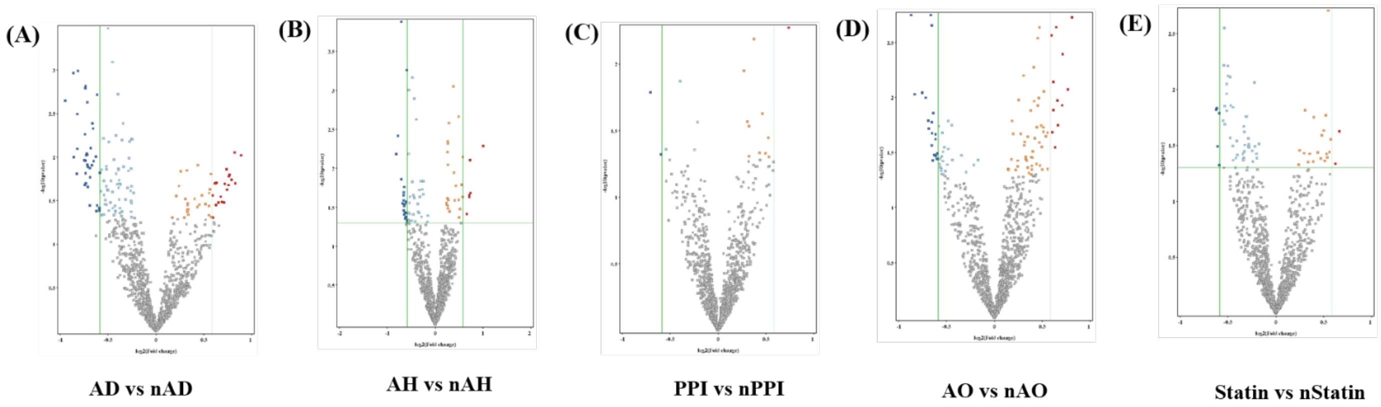

Figure S2: Dysregulated lipids as an effect of different medications taken by LBMD patients

(A-E) Volcano plots analysis show the dysregulated lipids as an effect of group of medications taken by LBMD patients, using a moderate t-test and considering fold change (FC) 1.5 with cutoffs of 0.05 (p-value). (A) Anti-diabetic medications (AD) associated with dysregulation of 57 lipids, (38 were down and 19 were up-regulated) (B) Anti-hypertensive medications (AH) associated with dysregulation of 28 lipids, (21 were down and 7 were up-regulated) (C) Proton pump inhibitors (PPI) associated with dysregulation of 3 lipids, 2 were down and 1 was up-regulated. (D) Anti-osteoporotic medications (AO) were associated with 32 lipids, 20 were down and 12 were up-regulated. (E) Anti-hyperlipidemic (statins) associated with dysregulation of 7 lipids, 5 were down and 2 were up-regulated).
